# Evaluation of rodent control to fight Lassa fever based on field data and mathematical modelling

**DOI:** 10.1080/22221751.2019.1605846

**Published:** 2019-04-21

**Authors:** Joachim Mariën, Benny Borremans, Fodé Kourouma, Jatta Baforday, Toni Rieger, Stephan Günther, N’Faly Magassouba, Herwig Leirs, Elisabeth Fichet-Calvet

**Affiliations:** a Evolutionary Ecology Group, University of Antwerp, Antwerp, Belgium; b University of California Los Angeles, Los Angeles, CA, USA; c Interuniversity Institute for Biostatistics and Statistical Bioinformatics (I-BIOSTAT), Hasselt University, Hasselt, Belgium; d Laboratoire des Fièvres Hémorragiques, Nongo, Guinée; e Bernhard-Nocht-Institute for Tropical Medicine, Hamburg, Germany

**Keywords:** Lassa virus, *Mastomys natalensis*, rodent control, rodent vaccination, arenavirus, rodent-borne virus

## Abstract

The Natal multimammate mouse (*Mastomys natalensis*) is the reservoir host of Lassa virus, an arenavirus that causes Lassa haemorrhagic fever in humans in West Africa. Because no vaccine exists and therapeutic options are limited, preventing infection through rodent control and human behavioural measures is currently considered to be the only option. In order to assess the efficacy of rodent control, we performed a 4-year field experiment in rural Upper Guinea and developed a mathematical model to simulate different control strategies (annual density control, continuous density control, and rodent vaccination). For the field study, rodenticide baits were placed each year in three rural villages, while three other villages were used as controls. Rodents were trapped before and after every treatment and their antibody status and age were determined. Data from the field study were used to parameterize the mathematical model. In the field study, we found a significant negative effect of rodent control on seroprevalence, but this effect was small especially given the effort. Furthermore, the rodent populations recovered rapidly after rodenticide application, leading us to conclude that an annual control strategy is unlikely to significantly reduce Lassa virus spillover to humans. In agreement with this finding, the mathematical model suggests that the use of continuous control or rodent vaccination is the only strategy that could lead to Lassa virus elimination. These field and model results can serve as a guide for determining how long and frequent rodent control should be done in order to eliminate Lassa virus in rural villages.

## Introduction

Lassa fever is a viral haemorrhagic fever caused by Lassa arenavirus (LASV), which is endemic in West Africa [1]. The main reservoir of the virus is the Natal multimammate mouse (*Mastomys natalensis*), a rodent species that thrives in rural villages and agricultural habitats, where it sheds the virus in its droppings and urine [2,3]. Humans mainly become infected through ingestion of contaminated food or water, inhalation of aerosolized virus particles, or direct consumption of an infected rodent [4,5]. Human-to-human transmission is thought to be rare and mainly occurs in households and hospitals [6,7]. By extrapolation from a seroepidemiological survey from 1987, it is estimated that between 100,000 and 300,000 infections could occur each year, with a fatality rate of 1–2% [8]. However, recent incidence reports suggest a substantial increase in the number and geographical extent of cases, exemplified by an unprecedented 2017–2018 outbreak in Nigeria [9,10]. Although this can partly be explained by the availability of better diagnostic tools and increased public awareness (especially after the 2014–2016 West Africa Ebola epidemic), increased spillover rates and transmission are likely, and can be driven by changes in climate, land use, and human mobility [11,12]. Because of the absence of a human vaccine or efficacious drug, the World Health Organization added LASV to its list of priority pathogens of epidemic potential for which there are no, or insufficient, countermeasures. The options for LASV prevention are currently limited to rodent control and changes in human awareness and behaviour [13,14]. Here, we investigate the feasibility of rodent control for managing the spillover of LASV from rodents to humans.

Rodent control is expected to reduce LASV spillover risk based on two independent assumptions: (i) the contact rate between rodents and humans is positively related to rodent population density and (ii) LASV prevalence in rodents is positively related to rodent population density [15]. We consider the first assumption to be valid, as long as there is no direct recolonization of houses. The second assumption is a consequence of host and virus characteristics. For directly transmitted microparasites, two contrasting transmission modes are typically considered: density-dependent transmission when host contact rate (and transmission) increases linearly with host density and frequency-dependent transmission when host contact rate remains constant regardless of density [16]. For parasites with density-dependent transmission, a density threshold is predicted below which the parasite cannot invade in the population [17,18]. Rodent control measures, aiming at reducing the density of rodents below this threshold, would then be effective for LASV elimination even if it does not lead to complete elimination of the rodents [15]. In contrast, parasites with frequency-dependent transmission are predicted to persist even in low-density populations, and (rodent) control measures aimed at reducing densities would be useless for LASV elimination. In this case, viral extinction can only be achieved if the proportion of susceptibles is too low for viral transmission (e.g. because most animals have become immune due to vaccination) [19,20].

For wildlife diseases, it is often difficult to determine direct relationships between parasite prevalence and host density because the ecology of the host is not well known [19,21]. However, as *M. natalensis* is the most important rodent pest species in sub-Saharan Africa, its ecology has been researched intensively [22–24]. *Mastomys natalensis* has a promiscuous mating system and is not territorial or aggressive towards conspecifics [25], and two studies independently found evidence for a strong positive relationship between population density and contact rates [26,27]. Furthermore, the analysis of a 10-year capture–mark–recapture time-series of a population of *M. natalensis* in Tanzania found that the transmission of Morogoro virus, an arenavirus genetically closely related to LASV, is probably density dependent [28]. It therefore seems safe to assume that the transmission of LASV in *M. natalensis* is density dependent.

As density dependence implies the existence of a density threshold for viral transmission, we expect that a reduction in rodent density will decrease and eventually prevent LASV transmission in the rodent population. In this study, we evaluate the effectiveness of this approach in rural villages in West Africa. First, we performed a 4-year field experiment in Upper Guinea, in which rodents were eliminated annually in three villages using rodenticides, while three other villages were used as control. Rodent seroprevalence and age distribution were monitored during the entire experiment. Using information obtained from this field experiment we then parameterized a mathematical model to simulate LASV transmission. The model was used to estimate the effect of different control strategies (annual density control, continuous density control, and rodent vaccination) on LASV invasion and extinction probability. The outcomes of the models can serve as a guide for how long and frequent rodent control should be done in order to eliminate LASV in a rural village.

## Methods

### Field experiment

#### Study sites

The field experiment was performed in the prefecture of Faranah (Upper Guinea), which was chosen for its high mean human LASV seroprevalence (35%) and the abundant presence of *M. natalensis* in the houses (>95% of captures is *M. natalensis*) [29,30]. In this area, six rural villages were selected based on the presence of LASV (seroprevalence in the rodent population >20%), their remote location from a paved road, a size not exceeding 1000 inhabitants and less than 45 min driving time from Faranah [31]. Rural villages in this area typically consist of groups of houses clustered within small agricultural or fallow land patches, which is optimal habitat for a commensal species such as *M. natalensis* [14]. The villages themselves lie within a matrix of tropical dry forest (within or closely located to the National Park of the Upper Niger), in which *M. natalensis* is absent [32]. This means that we can consider the villages to be effective islands in which *M. natalensis* can thrive, connected only by human traffic routes. The six villages were randomly grouped into control (Brissa, Dalafilani, and Yarawalia) and treatment (Damania, Sokourala, and Sonkonia) villages.

#### Rodenticide treatment

Rodenticide treatment was performed once a year over a period of 4 years (for 10 days during first 2 years and 30 days the last 2 years). The interventions were carried out during the dry season (November–April) when rodents were assumed to aggregate in houses to search for food and shelter. Anticoagulant baits (Bromadiolone or Difenacoum) were distributed in baiting stations (Coral, 158 Ensystex Europe) and were both placed in each in use house of the village, resulting in 300–600 baiting stations per village. We refer to Sáez et al. [31] for a more detailed explanation of the intervention and its effect on rodent abundance.

#### Rodent trapping

Rodents were trapped during three consecutive nights using Sherman live traps (Sherman Live Trap Co. Tallahassee, FL, USA), which were placed in pairs in 60 houses that were randomly chosen along a transect in the village. Traps were baited (with a mixture of peanuts, dry fish and wheat flour) in the evening and checked the next morning. Trapped rodents were humanely killed and necropsied in situ according to a biosafety protocol [23,33]. Blood was drawn from the hart with a syringe and preserved on prepunched filter paper (±15 µL/punch; Serobuvard, LDA 22, Zoopole, France). Eyes were preserved in 10% formalin. Trapping sessions were performed before and after intervention in the treatment villages and once a year in the control villages. Due to personnel safety issues, it was not possible to trap rodents in the villages Sokourala (years 2 and 3) and Sonkonia (year 2) during the Ebola epidemic [31]. In total, we analysed 14,394 trap nights. Trapping data are available at doi:10.6084/m9.figshare.5545267.

#### Serology

Filter paper was stored in small re-sealable zipper bags with desiccant silica gel at −20°C. Dried blood spots were punched out of the filter paper and eluted in phosphate buffer saline and 0.25% NH_3_ [34]. Presence of anti-LASV IgG antibodies in this elution was examined by indirect immunofluorescence assay [35,36]. Mouse antibodies were visualized using polyclonal rabbit anti-mouse IgG-FITC secondary antibodies (Dako, Denmark).

#### Eye lens weight

The age of individuals was estimated using eye lens weight (ELW), a known proxy for age in small mammals [37]. Eye lenses were extracted with forceps, cleaned, dried for 2 h at 100°C and weighed to the nearest 0.1 mg [36]. Raw ELW data were used as an age proxy for the statistical analysis of the field data. In order to parametrize the demographic component of the mathematical models (see below), we estimated age using the published function: age = e^(10.46088 + ELW/2)/4.35076 [22]. This conversion was not used for the statistical analyses because the estimate error becomes very large for ELW values above 25 mg.

#### Statistical analyses

In order to assess the population recovery rate after intervention, we tested the effect of rodenticide treatment on ELW distribution in the villages. For this analysis, we used a linear mixed model with treatment status (treatment vs. control village) and year (one to four as a factor) as independent fixed effects, village as random effect and ELW as dependent variable.

We were also interested to see whether rodenticide treatment affects the force of infection (FOI) in the rodent population. The FOI is an important epidemiological parameter that expresses the rate at which susceptible individuals become infected. Under the assumption of lifelong immunity, this parameter can be derived from age-specific seroprevalence data. In order to estimate FOI and test whether it is affected by treatment, we fitted generalized linear mixed models with ELW (proxy for age), treatment and year as fixed independent variables, village as random effect and antibody status (positive or negative) as dependent variable, assuming a binomial distribution with logit-link function. For this analysis we removed the youngest individuals from the dataset (ELW < 15 mg) because antibodies in these young animals could have been maternal.

We used the lmer and glmer functions of the lme4 package (version 1.1-7) of the R statistical software version 3.3.0 [38]. When fitting the models, we started with the fully parameterized models (all two-way interactions between the independent fixed variables) and sequentially dropped variables that had the highest insignificant *p*-values.

### Modelling LASV transmission

**Figure 1.**
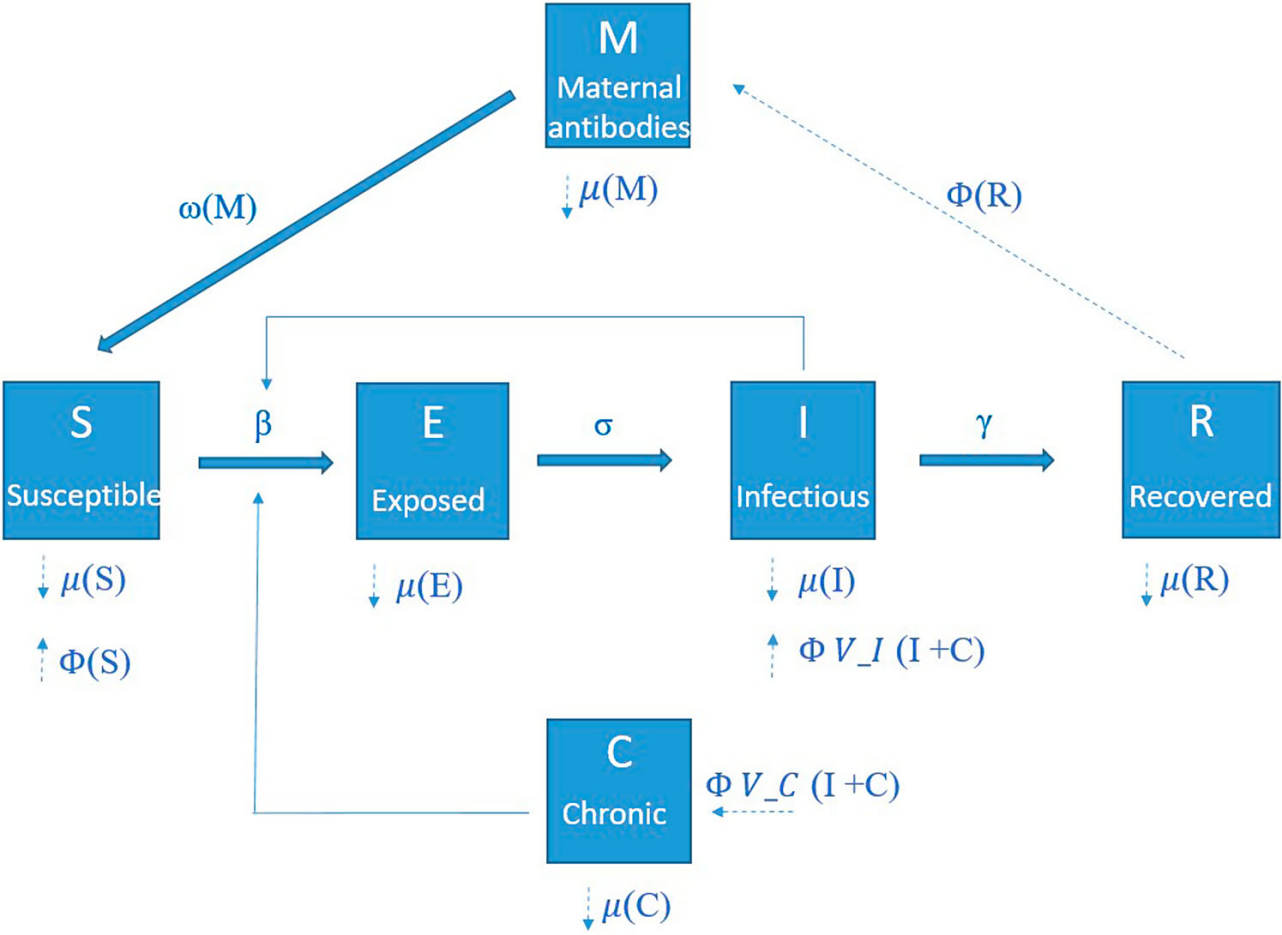
Schematic illustration of the individual-based model used to simulate the spread of Lassa virus in populations of *M. natalensis* in Guinea. Individual rodents are assigned different states according to infection status: susceptible (S), exposed (E), acutely infectious (I), recovered (R), maternal antibody positive (M), and chronically infectious (C). State transition rates depend on the following parameters: transmission coefficient (*β*), latent period (*σ*^−1^), infectious period (*γ*^−1^), maternal antibody period (*ω*^−1^). Fat solid arrows indicate possible transitions between different states. The dashed lines show the demographic parameters: Φ (birth rate) and μ (mortality rate). The probability to become acutely infected after vertical transmission is given by V_I and to become chronically infected by V_C. Thin solid arrows indicate that the rate at which individuals move from one state to another depends on the number of individuals in another state.

Using data from the field experiment and previous studies, we parameterized a stochastic individual-based model (IBM) to simulate the spread of LASV in a population of *M. natalensis* in Upper Guinea. The central aim of the modelling study was to investigate the effectiveness and sustainability of different control methods (annual density control, continuous density control, or rodent vaccination) to eliminate LASV from a rural village. The IBM is illustrated in Figure 1. Individuals are categorized in six compartments: susceptible (S), exposed but not infectious (E), acutely infectious (I), recovered (R), maternal antibodies (M), and chronically infectious (C). Both demographic and transition (movement of individuals between states) events were a function of time (unit of time is 1 day) and stochastic. For a full description of the model's demographic and transition parameters, we refer to the supplementary information (file: S.I. model).

#### Horizontal transmission component

Transmission in this study can be divided into horizontal and vertical components. Horizontal transmission of LASV occurs with an infection rate $\frac{\beta Sk^{q}\left(I+C\right)}{N^{q}}$, following the implementation of Smith et al. [39]. This formulation allows to easily compare the different shapes of the transmission–density relation by adjusting the parameter *q*: if *q* = 1, transmission is independent of density (frequency dependence); if *q* = 0, transmission is linearly related to density (density dependence); and if 1 > *q* > 0, transmission follows a power function (intermediate between frequency- and density-dependence). Because contacts of *M. natalensis* increase significantly with density [27], we suggested that *q* is (close to) zero for transmission of MORV in *M. natalensis*. However, as the commensal *M. natalensis* populations in West Africa might differ from the wild populations in East Africa, we implemented four different *q* values during the model simulations (*q* = 0, 0.25, 0.50, 0.75). The parameter β represents the transmission coefficient, which is composed of k (the contact rate at a given q) and v (probability of transmission between an infectious and a susceptible individual if they make a contact), and can be derived from the FOI (β = FOI/I). Optimization of β was done by comparing the FOI of the model to the field experiment (age and seroprevalence data from trapping sessions before intervention) for different values of *q*. Given that *Mastomys* is not territorial and seems to move randomly across the (relatively small) villages [14], we assumed a homogeneously mixing community in which all individuals are also identical with respect to susceptibility and infectivity. Vertical transmission is described in detail in the supplementary information (S.I. model).

#### Implementation of control methods

We investigated three potential LASV control methods:

**Annual density control** represents a situation similar to the interventions of the field experiment. The rodent population was reduced once a year by random removal of individuals in the model. The population then completely recovered within ten months after the intervention because of a birth rate (Φ) increase, which was included in the model. We assumed that the complete elimination or rodents in a village was unlikely (e.g. rodents that live outside or in closed houses) and therefore considered a 90% reduction the most realistic limit.

**Continuous density control** represents a situation in which the average rodent density (N_d_) is reduced for a long period. This could be implemented by continuously distributing rodent traps or poison in houses and proximate cultivations, storing food into rodent-proof containers, or attracting more domestic or wildlife predators to the village. This situation was simulated by reducing N_d_ of the model over a period of 10 years.

**Rodent vaccination** represents a situation in which animals become immune due to an oral vaccine. This situation was simulated by changing the infection status of a random subset (50–90%) of susceptible individuals (S) to recovered (R). These different scenarios were simulated for the distribution of an oral vaccine between once and four times per year.

For all control methods, we investigated whether they could eliminate LASV from the rodent population, how long (years) this would take and what the required mortality and vaccinated rate would be. We considered an elimination successful if the infection went extinct from the population within 11 years after the start of a simulation. We also investigated the **invasion probability** of LASV in a completely susceptible rodent population. An invasion was considered successful if the seroprevalence was higher than 10% at any moment after one infectious individual entered the population. We ran 1000 simulations for each combination of control method and transmission–density coefficient (*q* = 0, 0.25, 0.50, 0.75). The R-code of the model with additional information can be found in the supplementary materials.

## Results

### Field experiment statistics

**Figure 2.**
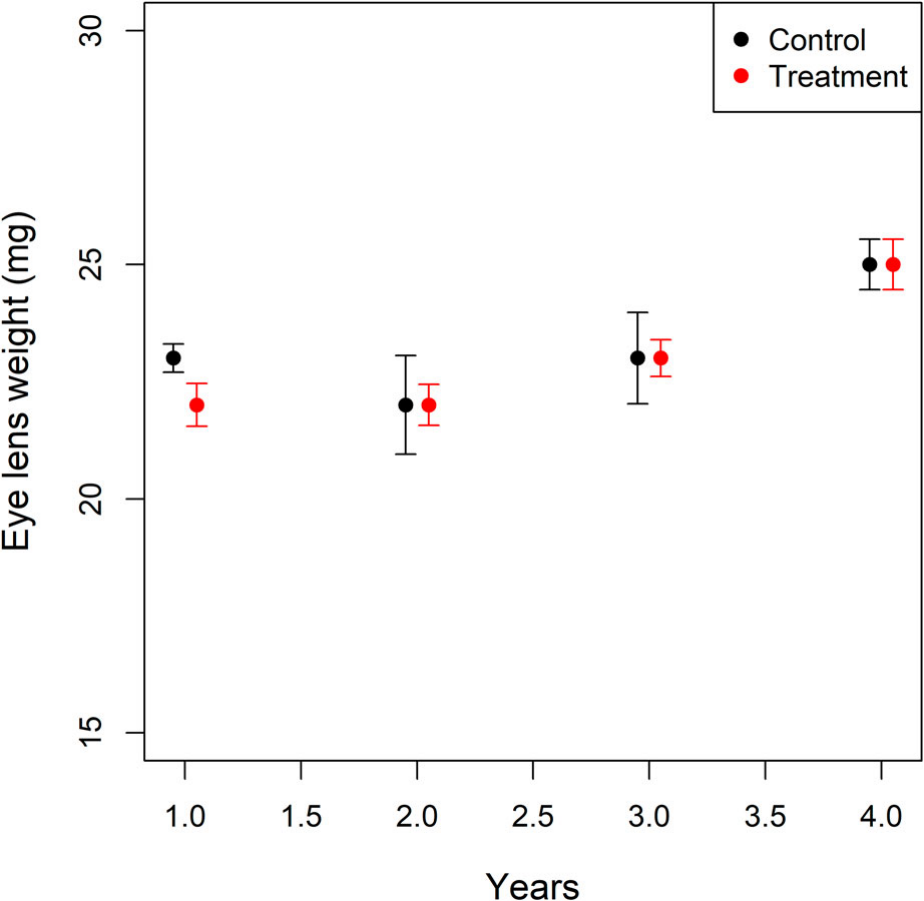
Mean eye lens weight (ELW) of *M. natalensis* for the control (black) and treatment villages (red) in function of year. The ELW is used as a proxy for age in the rodents. The error bars indicate standard errors on the means.

The trapping rate of *M. natalensis* was higher before than after rodenticide treatment (70–80% reduction), but there was no decrease in pre-treatment abundance over time (years) nor did we find a difference between treatment and control villages (supplementary figure 2 and [31] for *P*-values). In accordance, the mean ELW of pre-treatment individuals did not differ significantly between treatment and control villages (*χ*^2 ^= 0.89, df = 1, *p* = 0.34) and actually increased over time in both types of villages (*χ*^2 ^= 26.58, df = 1, *p* < 0.0001) (Figure 2).

**Figure 3.**
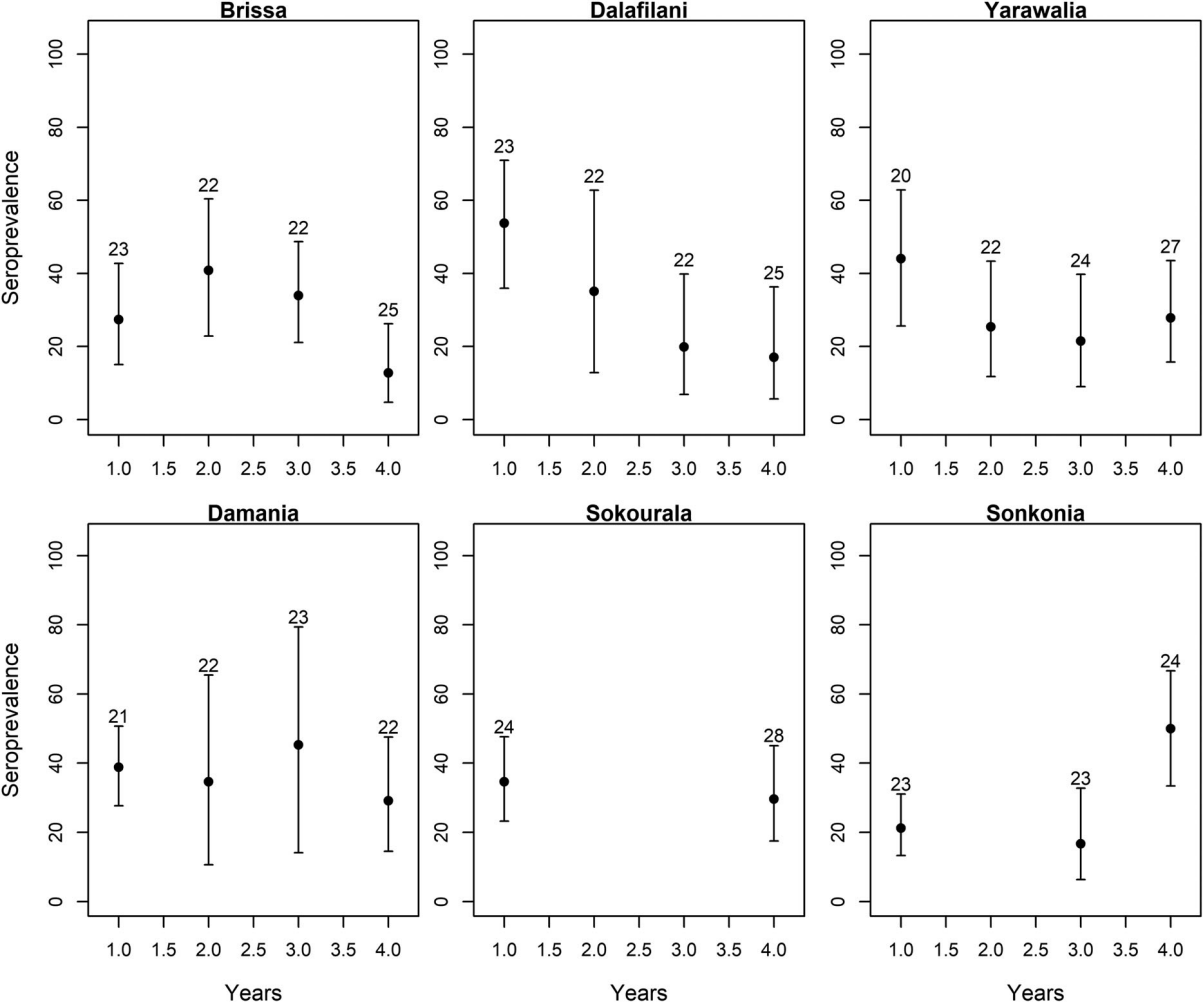
Mean LASV seroprevalence of *M. natalensis* in the treatment (Brissa, Dalafilani, and Yarawalia) and control (Damania, Sokourala, and Sonkonia) villages over time (years). The seroprevalence was corrected by the mean eye lens weight (number on top of the bars in mg), a known proxy for age in mammals. Bars indicate 95% (binomial) confidence intervals on the mean seroprevalence.

Antibody presence increased significantly with rodent age (*χ*^2 ^= 71.30, df = 1, *p* < 0.0001) with an interaction between treatment and time (*χ*^2 ^= 15.69, df = 1, *p* < 0.0001), where seroprevalence decreased over time in the treatment villages (reduction of 5% per year over the whole age distribution), while remaining constant in the control villages (supplementary figure 3). We also observed that the average seroprevalence (corrected for age effects using the slope of the ELW∼seroprevalence correlation in year 1) decreased over time in all treatment villages, but not in the control villages (Figure 3).

### Simulation results

#### Annual rodent control

**Figure 4.**
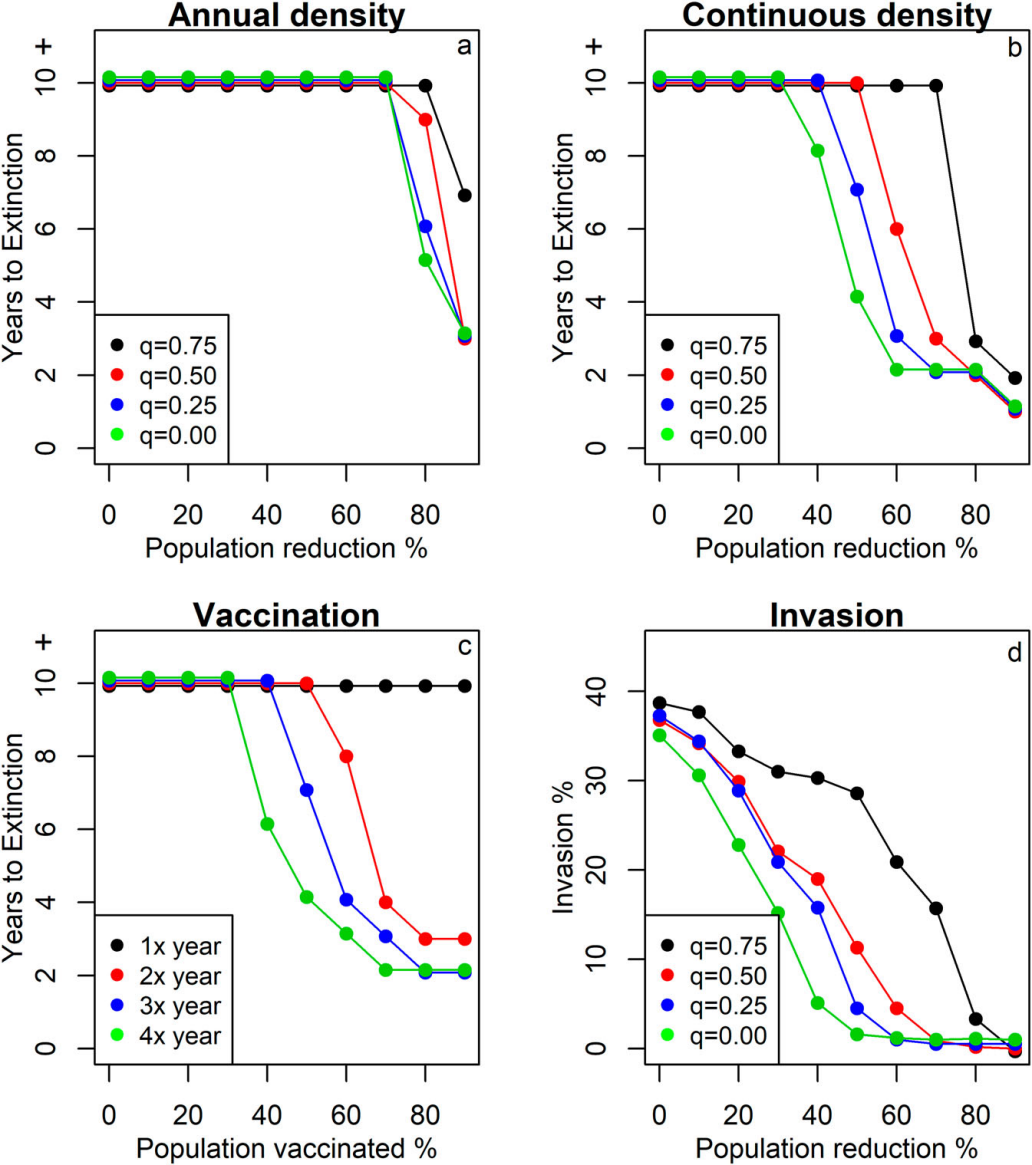
Model simulations to predict the effect of rodent control on LASV extinction probability in a population of *M. natalensis* in a rural village in Upper Guinea. The figures a, b and c show the number of consecutive years that rodents need to be controlled/vaccinated to ensure LASV extinction (>95% of simulation extinct). If “years to extinction” equals 10 years, at least 10 years or more will be necessary to ensure extinction. Figure d shows the invasion probability of LASV when one LASV positive *M. natalensis* enters a completely susceptible population in a rural village. The different colours represent simulations at different values of the transmission-density coefficient (*q* = 0 is density-dependent transmission; *q* = 1 is frequency-dependent transmission) or times that rodents were vaccinated per year (when *q* = 0.25).

For annual rodent control, the model suggests that viral extinction only occurs if the population density is reduced by at least 60% for ten consecutive years (supplementary figure 4a). However, the extinction probability strongly depends on the transmission–density coefficient (*q*). For example, if 60% of the population is killed each year, the predicted extinction probability varies from 1% for *q* = 0.75 (slight density dependence) to 85% when *q* = 0 (full linear density dependence). If we assume that the true value of *q* is around 0.25 (based on MORV data) [28], annual rodent control is predicted to ensure LASV elimination (i.e. extinction probability ≥95%) only if more than 80% of the rodents are eradicated for a period of at least eight years (Figure 4a).

#### Continuous rodent control

In comparison with annual control, continuous control is more effective at eliminating LASV from the rodent population. Although the extinction probability again strongly depends on the assumed transmission–density coefficient (*q*), viral extinction can already be achieved at 30% population reduction (*q* < 0.75) in 10 years (supplementary figure 4b). If we again assume that *q* = 0.25, rodent control is predicted to ensure LASV extinction when densities are reduced by 50% for 7 years (Figure 4b).

#### Rodent vaccination

The simulations predict that a rodent vaccine could effectively eliminate LASV if it is distributed more than once per year and when more than 60% of rodents become vaccinated (supplementary figure 4c). For example, if the vaccine is distributed three times per year and 60% is vaccinated, viral extinction is predicted to happen within four years, regardless of which *q* is assumed (as rodents densities remain constant in this scenario) (Figure 4c). In contrast, viral extinction can never be ensured when the vaccine is distributed only once per year, not even at high vaccination rates (90%).

#### Simulation results – invasion probability

LASV invasion probability increased positively with population density and the transmission–density coefficient (*q*). For *q* = 0.25, successful LASV invasion can be expected at population densities reduced by 60% (population density of 32 *Mastomys*/ha) or less. At higher values of *q* (i.e. larger contribution of frequency dependence), the population would need to be reduced by at least 90% to prevent LASV invasion (Figure 4d).

## Discussion

We hypothesized that rodent control can be used to reduce LASV spillover risk to humans through a decrease in rodent density and/or LASV prevalence in the rodent population. Our field experiment shows that an annual rodenticide treatment can indeed strongly reduce rodent density in a rural village, but also that the population quickly returns to levels equivalent to those of the previous year ([31] and ELW data). We also found that annual control can reduce LASV prevalence in the rodent population, but this reduction is likely too small (±5% per year) to be a cost-effective and feasible strategy, given the high workload and financial costs. After 4 years of rodent density control, we can conclude that annual control is unlikely to significantly reduce LASV spillover risk to humans, as both rodent density and LASV prevalence rapidly return to pre-treatment levels. The inability to eliminate a sufficiently large proportion of the population (only rodents in houses) and the rapid recolonization and birth rate of *M. natalensis* are the main reasons for the limited success [22]. Although the treatment effectively killed rodents in the houses (based on the carcasses that were found), we placed the baiting stations indoor and in open houses only. Rodents that lived outside or in closed houses remained unaffected and could continue breeding and transmitting the virus, before recolonizing the previously treated houses. In support of this reasoning, local villagers had the impression that rodents returned quickly after the treatment, especially in houses close to the village border [31].

Nevertheless, even though only a part of the total rodent population was eliminated, we did observe a small significant negative effect (5% per year) on seroprevalence. This suggests that LASV transmission is density-dependent and that a density threshold exists below which the virus cannot persist in the rodent population [15]. However, the mathematical model suggests that the threshold is low, probably due to a small subset of chronically infectious animals that can continue the transmission chain at low densities [40]. If rodent control is performed once per year, the model indicates that rodent densities need to be reduced by 80% for at least 8 years to ensure virus extinction. This would be very difficult to achieve given that rodent elimination would need to happen both indoors and outdoors, while it was already challenging to eliminate rodents in open houses only. Furthermore, if rodent control would stop after LASV extinction and rodent densities would return to pre-treatment levels, LASV could re-invade rapidly in the susceptible population (invasion probability is ±40% for even a single infectious rodent). Successful LASV invasion could be the result of an infectious rodent that arrives from a neighbouring village using human traffic routes (e.g. in food trucks) or could be the result of a reverse zoonosis (human infects rodent) [41]. The latter transmission route is not documented in the literature, but was earlier hypothesized to explain the transmission dynamics of the disease: humans become vectors by excreting the virus in urine and saliva on the ground, infecting rodents through contact with this contaminated soil [36].

In contrast to annual density control, continuous density control would be a more promising strategy for eliminating LASV from the rodent population. The model predicts that reducing rodent densities by 60% can ensure LASV extinction if it is maintained for at least four years. Continuous control however is labour-intensive and demands a human behavioural shift that includes the participation of the majority of the village community [42]. We recently proposed the development of an integrated control system by combining poisoning with regular trapping [31]. In this scenario, poisoning during the dry season would be combined with indoor trapping during the remainder of the year. In addition to active rodent control, specific changes in human behaviour could also reduce rodent densities, and are likely to be a more sustainable strategy. The hygienic state of houses in these rural villages is currently so low that even simple interventions (e.g. rodent proofing of houses or storing food in airtight containers) could have a large impact on indoor rodent abundance [5]. In contrast, outdoor control could be more difficult to implement. Outdoor rodenticide treatment is less advised due to the potentially negative effects on other wild or domestic animals, and because traps are often stolen [43]. An alternative for outdoor lethal control is fertility control through the distribution of hormones (or other chemical compounds) in baits [44]. This approach is not only suggested to be safer but also to be more cost effective and sustainable because it prevents compensatory reproduction and increased survival of rodents, which is often observed after rodenticide treatments [45–47]. Preliminary studies with synthetic steroid hormones (quinestrol and levonorgestrel) in wild *M. natalensis* in Tanzania show highly promising results [48]. In addition to lethal and fertility control, biological control (e.g. attracting predatory birds to the village) might also work outdoors and could have long-lasting effects, which are not dangerous for humans or domestic animals [49,50]. Currently, predatory bird densities are very low in these areas because villagers kill these animals for food and superstitious reasons (e.g. owls are believed to represent bad ghosts), which might contribute to high rodent densities.

We also assessed whether rodent vaccination could be an efficient alternative for eliminating LASV. The models suggest that this approach could work if it is performed more than once per year and more than 60% of the population is vaccinated during four years (which means that the vaccine must be consumed by the majority of the population and must be effective against the present LASV clade). Although an oral vaccine has not yet been developed, it might be easier and cheaper to produce than a human vaccine, as it will be possible to skip the expensive and time-consuming clinical test phases [51,52]. The vaccine could be distributed to rodents in the form of bait pellets, similar to how rodents consume rodenticide. According to our knowledge, no attempts have been made to vaccinate rodent populations, and the elimination of rabies in foxes is the only example where a vaccine was used to control a wildlife disease [20]. Nevertheless, the delivery of an oral vaccine has been proposed for other wildlife diseases, including chlamydial infection in koalas, Tasmanian devil facial tumour disease and chytridiomycosis in frogs [53–55].

The mathematical model was based on existing data, and although good data exists to inform the model parameters, the model can be improved by additional field data. First, the model could benefit from more information about *M. natalensis* densities in these rural villages, in order to know how many rodents need to be eliminated. Such quantitative information is available for many areas of sub-Saharan Africa, but not for the Lassa fever-endemic region [28,56]. Other important aspects that can be improved are the transmission-density relation and the percentage of chronically infected animals in the wild. Recent data about MORV in *M. natalensis* improved our understanding of arenavirus ecology, but additional studies on LASV are necessary to compare these results [28].

## Ethics statement

All experiments were approved by the National Ethics Committee of Guinea (permit n° 12/CNERS/12 and 129/CNERS/16), performed in collaboration with the local health authorities (Prefecture de Faranah) and in agreement with the village chiefs. Rodenticide and traps were only placed in a house if permission was obtained from the individual house owner.

## Supplementary Material

Supplemental Material
