# Supplementary material for: Evaluation of rodent control to fight Lassa fever based on field data and mathematical modelling
Source: Emerg Microbes Infect. 2019 Apr 21;8(1):640–9. doi: 10.1080/22221751.2019.1605846 (PMC7011821; doi:10.1080/22221751.2019.1605846)
Supplement: Supplemental Material [file TEMI_A_1605846_SM5522.zip › Supplementary information figures.docx]

**Supplementary Figures**


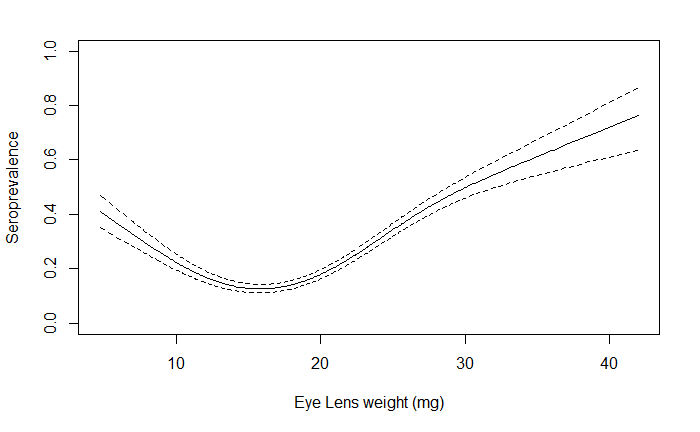


**Supplementary Fig 1:** Proportion of LASV antibody-positive *Mastomys natalensis* as a function of the eye lens weight (proxy for age) modelled by a generalized additive model. Antibodies of individuals younger than 15mg were considered to be derived from the mother.


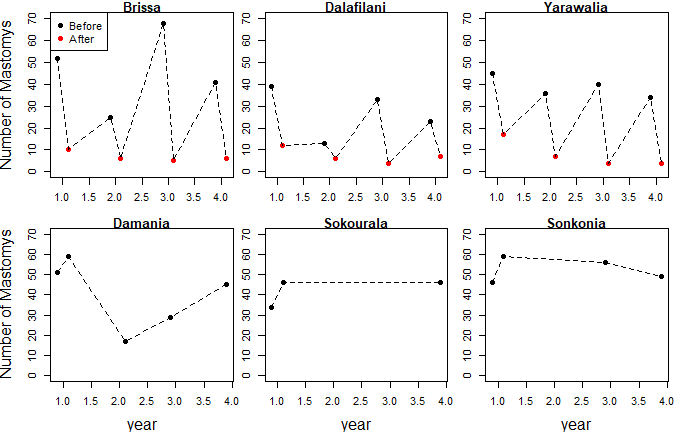


**Supplementary Fig 2:** Number of *Mastomys natalensis* captured per trapping session before (black) and after (red) rodenticide treatment [38].


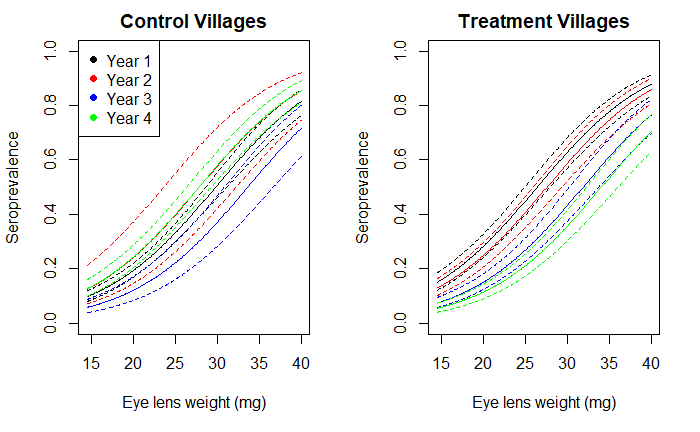


**Supplementary Fig 3:** Proportion of LASV antibody-positive *Mastomys natalensis* as a function of the eye lens weight (proxy for age) for the control (left) and treatment (right) villages. The different colours represent the different years when rodents were captured. The solid lines represent mean estimated seroprevalence values (derived from a generalized linear model) and dashed lines standard errors.


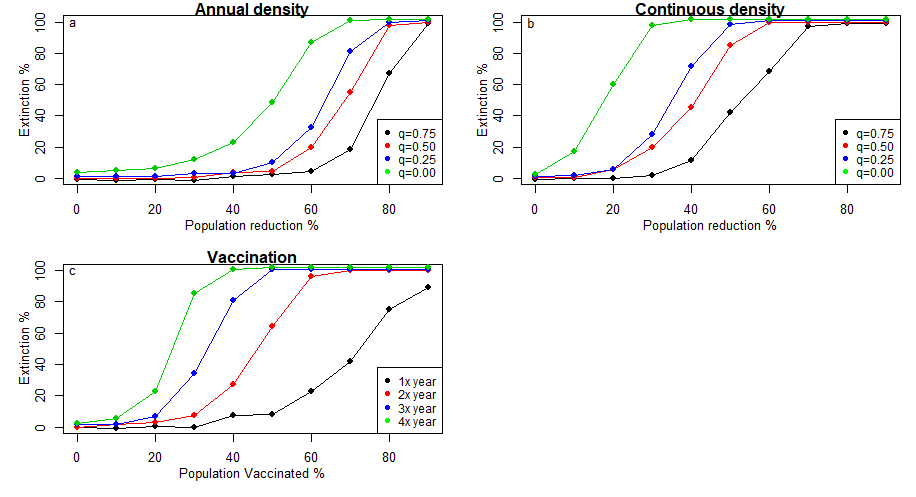


**Supplementary Fig 4:** The figures show the extinction probability in function of population reduction if (a) rodent control was performed annually; (b) rodent control was performed continuously; or (c) rodents were vaccinated for ten consecutive years. The different colours represent simulations at different values of the transmission-density coefficient (q=0 is density-dependent transmission; q=1 is frequency- dependent transmission) or times that rodents were vaccinated per year.
