# Supplementary material for: Evaluation of rodent control to fight Lassa fever based on field data and mathematical modelling
Source: Emerg Microbes Infect. 2019 Apr 21;8(1):640–9. doi: 10.1080/22221751.2019.1605846 (PMC7011821; doi:10.1080/22221751.2019.1605846)
Supplement: Supplemental Material [file TEMI_A_1605846_SM5522.zip › Supplementary information model.docx]

**Description model figure 1**

Using data from the field experiment and previous studies, we parameterized a stochastic individual-based model (IBM) to simulate the spread of LASV in a population of *M. natalensis* in Upper Guinea. The central aim of the modelling study was to investigate the effectiveness and sustainability of different control methods (annual density control, continuous density control, or rodent vaccination) to eliminate LASV from a rural village. The IBM is illustrated in Figure 1. Individuals are categorized in six compartments: susceptible (S), exposed but not infectious (E), acutely infectious (I), recovered (R), maternal antibodies (M), and chronically infectious (C). Both demographic and transition (movement of individuals between states) events were a function of time (unit of time is 1 day) and stochastic.

***Demographic component***

In order to test the impact of rodent control, the average rodent density in a typical rural village was implemented in the model. The best possible estimations on *M. natalensis* densities in Upper Guinea can be derived from a capture-mark-recapture study, indicating a rough estimate of 80 *M. natalensis* per hectare over the course of one month in a rural village ^1^. Another field study showed that the overall trapping rate remained constant over the years ^2^. Based on these studies, we considered 80 *M. natalensis* per hectare to be a realistic average density (*N_d_*) and assumed that it remains constant over time. To estimate the total rodent population size in a village, we measured the average area of villages in this region using google earth (±25ha) and multiplied it by the average rodent density (25ha x 80 mice/ha = 2000 mice per village).

It was also important to implement a realistic age distribution in the model, as age-specific seroprevalence was used to estimate the FOI of the model. Optimization of birth (Φ) and mortality (μ) parameters was done by comparing the age distribution of the model to the field data (ELW data from trapping sessions before intervention). Birth (Φ) was a function of density (*N*) and modelled using the following equations:

Φ (N > 0.75* N_d_) = 0.0027 [births/(individual*time interval)]

Φ (N ≤ 0.75*N_d_)= 0.0044 [births/(individual*time interval)].

If host density was below 0.75*(*N_d_*) (e.g. after intervention), we increased the birth parameter so that the population would recover at the same rate as observed in the field experiment. Age of individuals followed an exponentially decreasing distribution based on the mortality parameter μ, which was age dependent and modelled by these equations:

μ (age < 50) = 0.002 lifespan^-1^

μ (49 < age < 100) = 0.003 lifespan^-1^

μ (99 < age < 366) = 0.007 lifespan^-1^

μ (age > 365) = 0.02 lifespan^-1^.

Because arenaviruses have no or limited adverse effects on their reservoir hosts, we assume that the birth and death rates are unaffected by infection ^3,4^, so infectious individuals have the same demographic parameters as other individuals in the model.

**Horizontal transmission component**

Transmission in this study can be divided into a horizontal and vertical component. Horizontal transmission of MORV occurs with an infection rate $\frac{\beta Sk^q(I+C)}{N^{q}}$, following the implementation of Smith et al. (2009). This formulation allows to easily compare the different shapes of the transmission-density relation by adjusting the parameter q: if q=1, transmission is independent of density (frequency-dependence); if q=0, transmission is linearly related to density (density-dependence); and if 1>q>0, transmission follows a power function (intermediate between frequency- and density-dependence). Because contacts of M. natalensis increase significantly with density ^27^, we suggested that q is (close to) zero for transmission of MORV in M. natalensis. However, as the commensal M. natalensis populations in West Africa might differ from the wild populations in East Africa, we implemented four different q values during the model simulations (q=0, 0.25, 0.50, 0.75). The parameter $\beta$ represents the transmission coefficient, which is composed of k (the contact rate at a given q) and v (probability of transmission between an infectious and a susceptible individual if they make a contact), and can be derived from the FOI ($\beta$=FOI/I). Optimization of $\beta$ was done by comparing the FOI of the model to the field experiment (age and seroprevalence data from trapping sessions before intervention) for different values of q. Given that Mastomys is not territorial and seems to move randomly across the (relatively small) villages ^14^, we assumed a homogeneously mixing community in which all individuals are also identical with respect to susceptibility and infectivity. Vertical transmission is described in detail in the supllementary information (S.I. model).

***Vertical transmission***

Vertical transmission occurs at the following infection rate: V_I [Φ (I+C) ] + V_C [Φ (I+C) ]. The model assumes that juveniles can enter the population as acutely (I) or chronically (C) infectious. The parameter V_C defines the proportion of juveniles that will become chronically infectious at birth. This parameter was fixed so that approximately 10% of the infectious individuals in the population became chronically infected, which matches findings of previous studies on LASV and MORV ^5–7^. These chronic carriers were assumed to stay infectious for the rest of their lives. The parameter $(V\_I$=1-V_C $)$defines the proportion of juveniles that will become acutely infectious by vertical transmission$.$

**Transition states**

The model assumes that when a susceptible individual becomes infected, it first passes a latency state (E) for an average of 4 days ($\sigma$^-1^) during which it is not infectious. Subsequently, it becomes acutely infectious (I) for an average of 35 days ($\gamma$^-1^), after which it recovers from the disease and develops lifelong immunity (R). The average latency and infectious lengths were derived from inoculation experiments of LASV and MORV in M. natalensis ^5,8^. We finally assume that offspring from antibody-positive mothers (R) will acquire maternal antibodies (M). These are present for an average of 30 days ${(\omega}^{-1})$, after which they disappear and the individual becomes susceptible. Direct evidence of maternal antibodies is not available for LASV in M. natalensis, but the decrease in seroprevalence from newborns to juveniles in the field data suggests that maternal antibodies indeed occur (supplementary figure 1; and ^9^). Furthermore, maternal antibodies were directly observed for lymphocytic choriomeningitis virus (LCMV) in laboratory experiments ^6,7,10^. The assumption that we make in the model is that every infected mouse remains in one state (uniform distribution with probability of transition 0) for a fixed amount of time before transitioning to a different state depending on a probability that follows the exponential distribution.

**
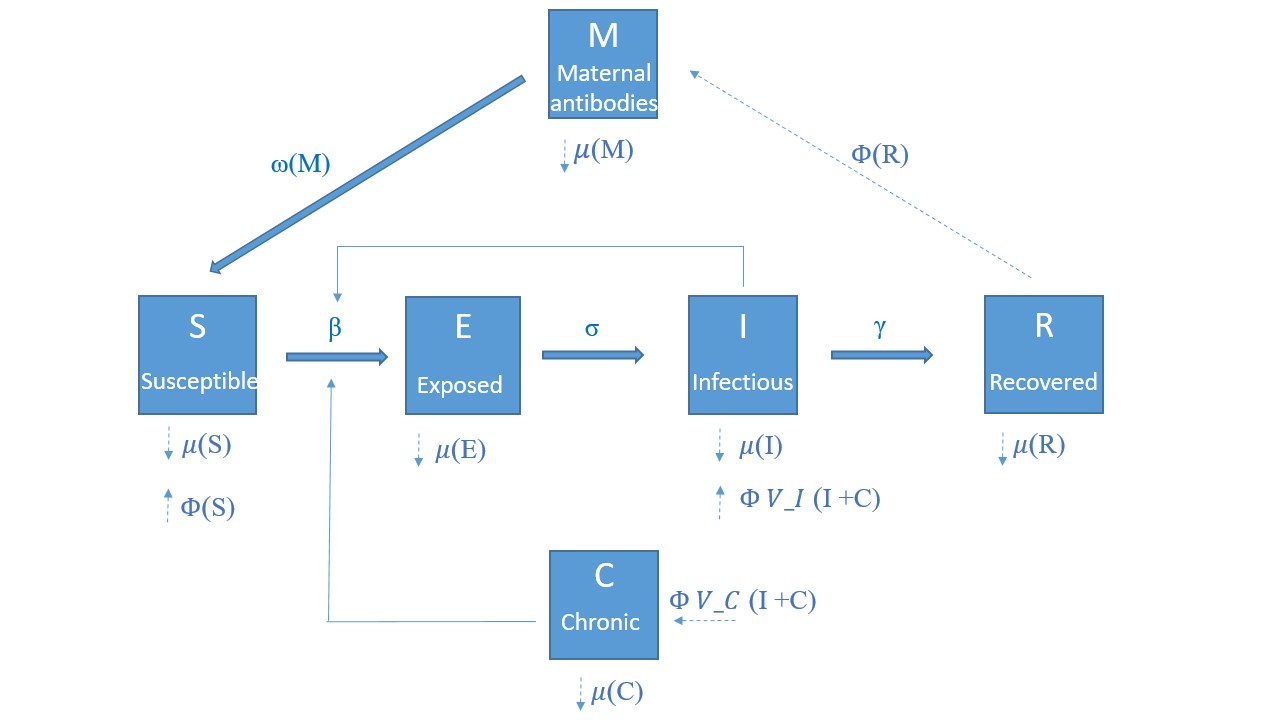
**

**Fig 1:** Schematic illustration of the individual-based model used to simulate the spread of Morogoro virus in populations of M. natalensis in Tanzania. Individual rodents are assigned different states according to infection status: susceptible (S), exposed (E), acutely infectious (I), recovered (R), maternal antibody positive (M) and chronically infectious (C). State transition rates depend on the following parameters: transmission coefficient (β), latent period (σ^-1^), infectious period (γ^-1^), maternal antibody period (ω^-1^). Fat solid arrows indicate possible transitions between different states. The dashed lines show the demographic parameters: Φ (birth rate) and μ (mortality rate). The probability to become acutely infected after vertical transmission is given by V_I and to become chronically infected by V_C. Thin solid arrows indicate that the rate at which individuals move from one state to another depends on the number of individuals in another state.

**References**

1. Mariën, J., Kourouma, F., Magassouba, N., Leirs, H. & Fichet-Calvet, E. Movement Patterns of Small Rodents in Lassa Fever-Endemic Villages in Guinea. *Ecohealth* **15,** 348–359 (2018).

2. Fichet-Calvet, E. *et al.* Fluctuation of abundance and Lassa virus prevalence in Mastomys natalensis in Guinea, West Africa. *Vector Borne Zoonotic Dis.* **7,** 119–28 (2007).

3. Mariën, J. *et al.* No measurable adverse effects of Lassa, Morogoro and Gairo arenaviruses on their rodent reservoir host in natural conditions. *Parasit. Vectors* **10,** 210 (2017).

4. Mariën, J. *et al.* Arenavirus infection correlates with lower survival of its natural rodent host in a long-term capture-mark-recapture study. *Parasit. Vectors* **11,** 90 (2018).

5. Walker, D. H., Wulff, H., Lange, J. V & Murphy, F. A. Comparative pathology of Lassa virus infection in monkeys, guinea-pigs, and Mastomys natalensis. *Bull. World Health Organ.* **52,** 523–34 (1975).

6. Fichet-Calvet, E., Becker-Ziaja, B., Koivogui, L. & Günther, S. Lassa serology in natural populations of rodents and horizontal transmission. *Vector Borne Zoonotic Dis.* **14,** 665–74 (2014).

7. Mariën, J. *et al.* Arenavirus Dynamics in Experimentally and Naturally Infected Rodents. *Ecohealth* **14,** 463–473 (2017).

8. Borremans, B. *et al.* Shedding dynamics of Morogoro virus , an African arenavirus closely related to Lassa virus , in its natural reservoir host Mastomys natalensis. *Nat. Publ. Gr.* 1–8 (2015). doi:10.1038/srep10445

9. Demby, A. H. *et al.* Lassa Fever in Guinea: II. Distribution and prevalence of Lassa virus infection in small mammals. *Vector borne zoonotic Dis.* **1,** 283–299 (2001).

10. Oldstone, M. *Biology and pathogenesis of lymphocytic choriomeningitis virus infection*. (Springer-Verlag, 2002).
